# Extramammary Paget Disease of Oral Mucosa: Case Report with Literature Review

**DOI:** 10.1007/s12105-024-01638-1

**Published:** 2024-04-24

**Authors:** Melad N. Dababneh, Danielle M. Bottalico, Keith M. Schneider, Michelle Moh, Ivan J. Stojanov

**Affiliations:** 1 Robert J. Tomsich Department of Pathology and Laboratory Medicine, Cleveland Clinic, Cleveland, OH USA; 2 Head and Neck Institute, Cleveland Clinic, Cleveland, OH USA; 3 Private Practice, Mentor, OH USA; 4 Department of Oral and Maxillofacial Surgery, Case Western Reserve University School of Dental Medicine, Cleveland, OH USA

**Keywords:** Paget disease, Extramammary, Oral mucosa

## Abstract

Extramammary Paget disease (EPMD) of the oral mucosa is an unusual and extremely rare condition, with fewer than ten cases documented. Here, we report a case of EMPD extensively involving oral mucosa and underlying salivary ducts in a 72-year-old male and review published clinical, histologic, immunophenotypic, and prognostic features of this rare entity.

## Introduction

Paget disease (PD) is an intraepithelial adenocarcinoma that classically involves the nipple-areolar epidermis, where it is termed mammary Paget disease (MPD) [1, 2]. MPD typically presents as an erythematous and/or scaly lesion that may be ulcerated or associated with nipple inversion or discharge. It accounts for 1–4% of breast carcinomas and is associated with underlying breast carcinoma in greater than 95% of cases. More rarely, PD may involve mucocutaneous surfaces such as of the anogenital region or the inguinal/axillary folds, where it is termed extramammary Paget disease (EMPD) [3–5]. In this setting EMPD may be primary, where its pathogenesis is incompletely understood, or secondary, where EMPD represents pagetoid involvement of anogenital skin by carcinoma originating from the lower gastrointestinal or genitourinary tracts.

Oral mucosal involvement by EMPD is extremely rare and has been documented, to the best of our knowledge, only eight times in the English-language literature over the last five decades [6–13]. Herein, we report a case of EMPD extensively involving the oral mucosa of an elderly patient and review the literature of this exceptional entity.

## Case Report

**Fig. 1:**
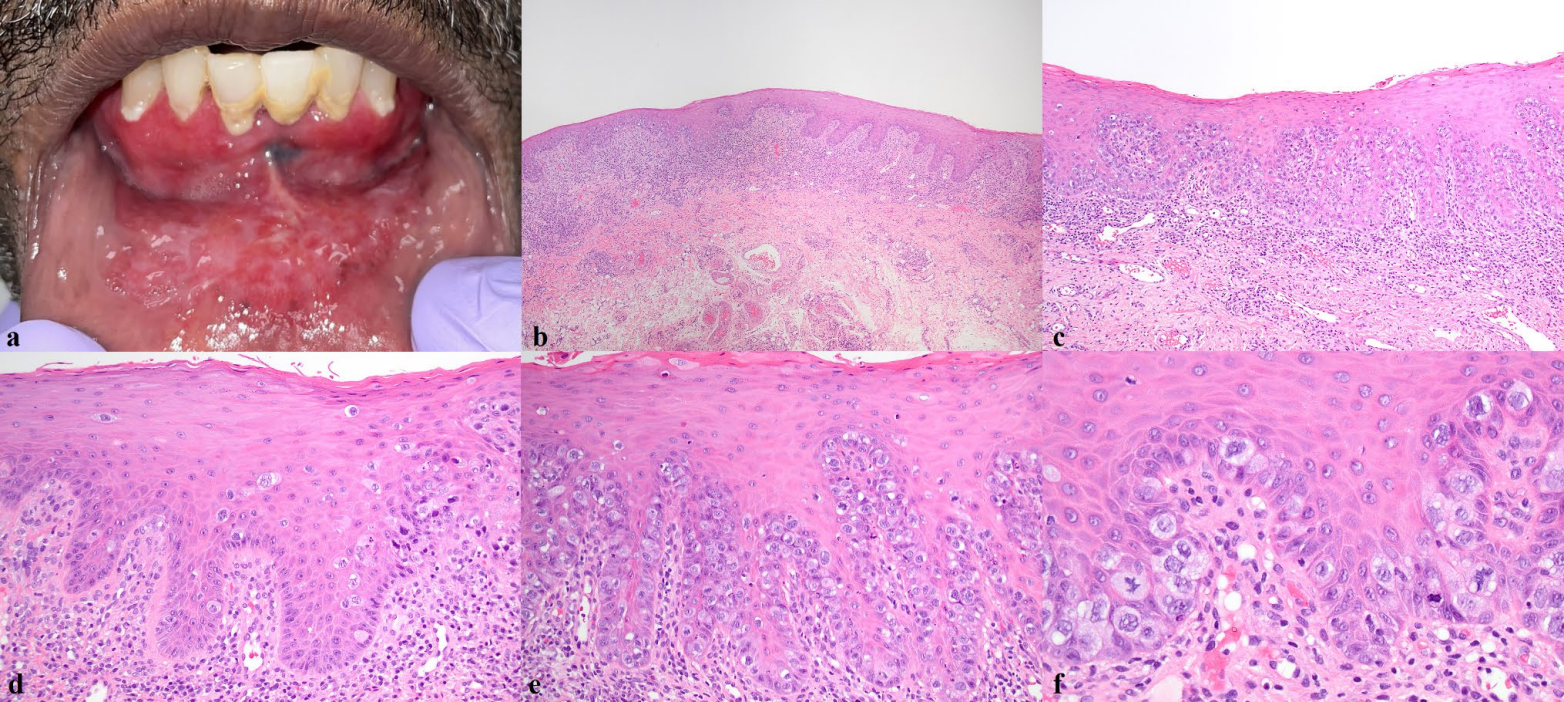
Extensive red/white mucosal change of anterior mandibular vestibule and lower lip concerning for lichenoid inflammatory process or dysplasia/carcinoma (**a**). Squamous mucosa with lymphocytic host response and atypical cells with amphophilic cytoplasm (**b**, ×40; **c**, ×400). Atypical cells primarily involve basal/parabasal layers but also demonstrate pagetoid involvement of spinous layer (**d**, ×200; **e**, ×200). Paget cells exhibit markedly atypical nuclei with abundant amphophilic cytoplasm (**f**, ×400)

A 72-year-old male smoker presented to a community-based oral and maxillofacial surgeon with unexplained weight loss of 9 kg over the last year and 1–2 months of anterior oral mucosal tenderness, most notable during eating, drinking, and toothbrushing. The intraoral examination was notable for red-and-white mucosal change diffusely involving the anterior mandibular vestibule, the facial and lingual gingiva of the anterior mandible, and the anterior floor of mouth (Fig. 1a). No discrete ulceration or masses were identified. The patient denied any significant past medical history including history of mucocutaneous disease or malignancy. The clinical impression was suggestive of lichen planus or dysplasia/squamous cell carcinoma, and a biopsy was performed.

The histologic findings were of squamous mucosa with epithelium extensively involved by atypical cells exhibiting abundant amphophilic cytoplasm and enlarged, hyperchromatic, and pleomorphic nuclei (Fig. 1b–f). A prominent lymphocytic infiltrate was present in the superficial lamina propria, resembling lichenoid mucositis or a host immune response to epithelial dysplasia.

**Fig. 2:**
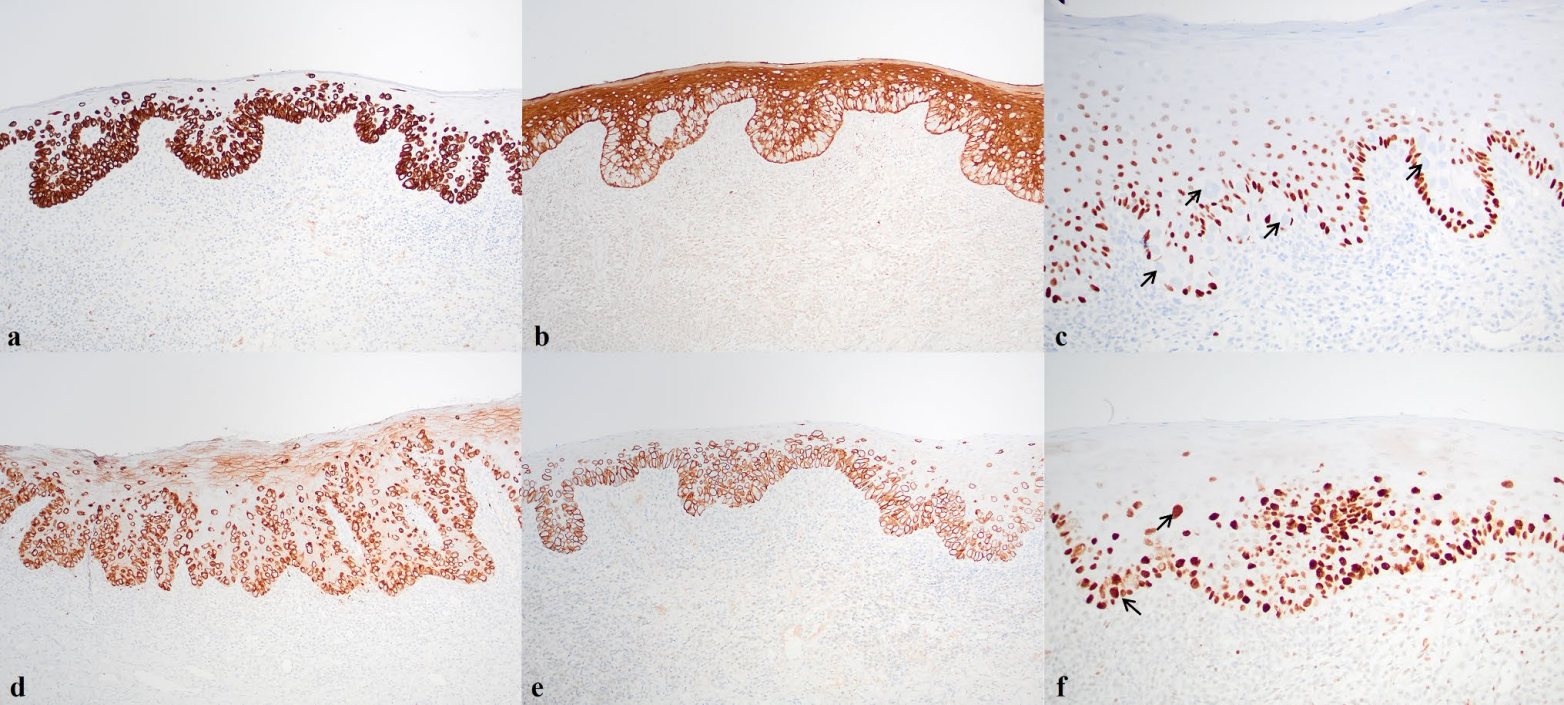
Paget cells were CK7 + (**a**, ×100) while CK5/6- (**b**, ×100) and p40- (**c**, ×200). Paget cells were additionally CEA + (**d**, ×100), Her2 3 + (**e**, ×100), and p53 + (**f**, ×200). Arrows highlight Paget cells in **c** and **f**

**Fig. 3:**
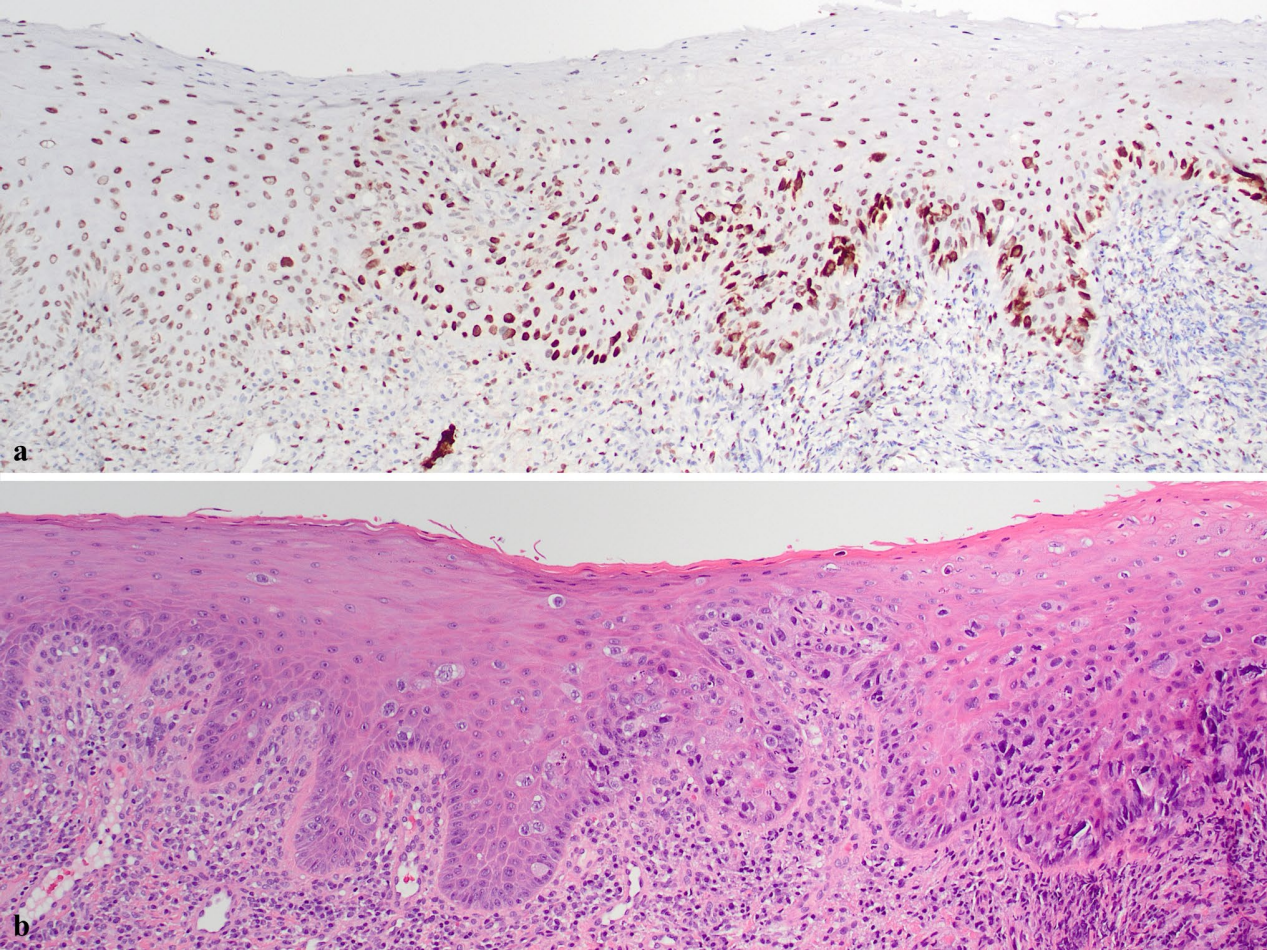
Paget cells were strongly positive for TRPS1 (right) while background normal stratified squamous epithelium (left) showed weaker nuclear positivity (**a** and **b**, ×100)

By immunohistochemistry (IHC), tumor cells were positive for CK7, CAM 5.2, EMA, CEA, TRPS1, Her2 (3 +), and mucin (focally), and showed a p53-mutant pattern of staining in the form of diffuse overexpression in tumor cells. Tumor cells were negative for CK5/6, p40, CK20, SOX10, p16, S100, and AR, with CK5/6 and p40 highlighting background surface epithelium (Figs. 2 and 3). Tumor was confined to epithelium and no salivary parenchyma was present in the biopsy specimen. The histologic and immunohistochemical features supported the diagnosis of EMPD.

**Fig. 4:**
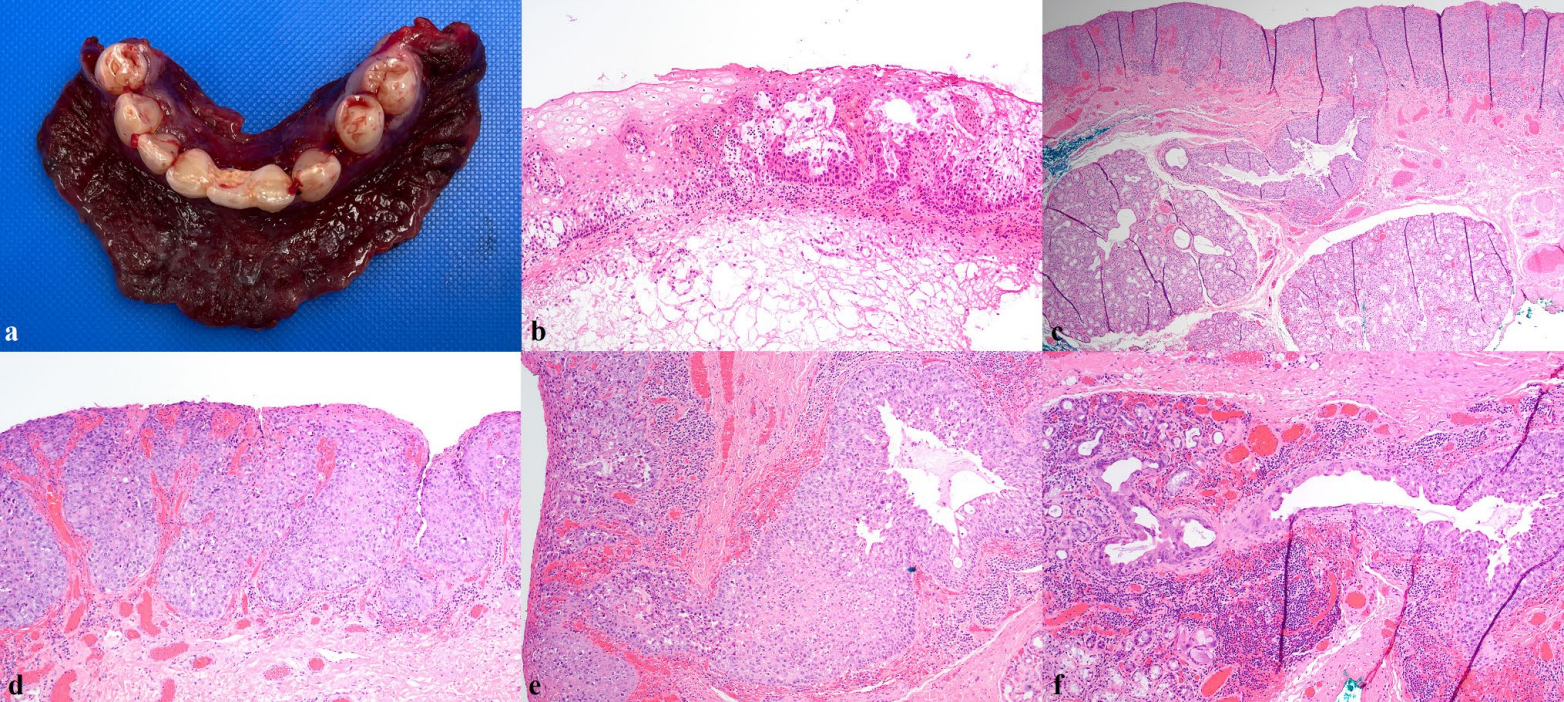
Frozen section evaluation of anterior mandible composite resection (**a**) with shave margin (**b**, ×100) focally involved by EMPD; uninvolved epithelium present at left. Resection specimen showed extensive involvement of surface epithelium and salivary excretory duct with no invasive adenocarcinoma (**c**, ×40; **d**, ×100). Salivary excretory duct showing continuity with surface epithelium and microscopic extension into minor salivary gland upon serial sectioning (**e**, ×100; **f**, ×200)

**Fig. 5:**
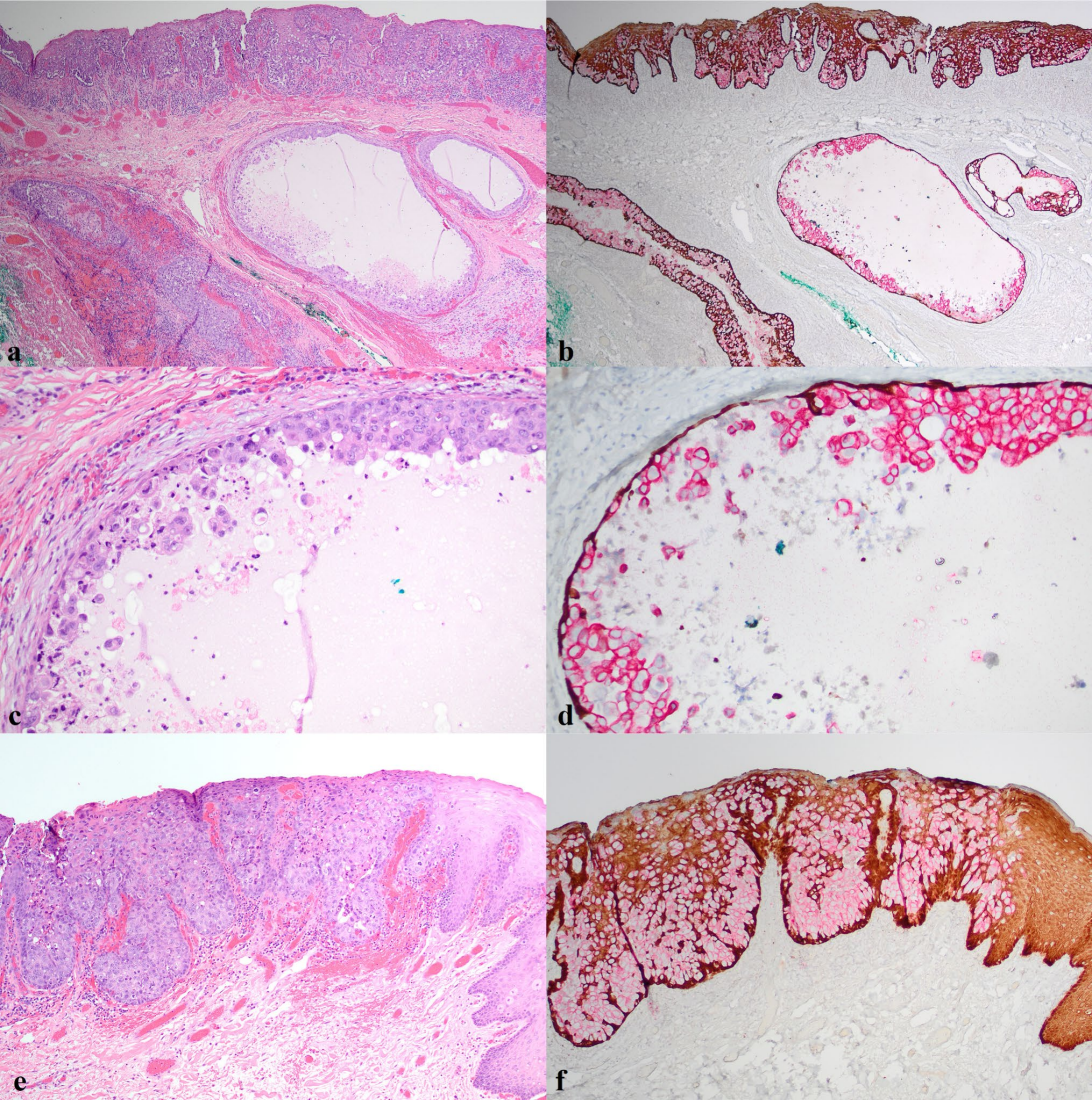
Extensive intraepithelial and intraductal involvement with no invasive adenocarcinoma (**a**, ×40; **b**, ADH5 ×40). Intraductal Paget cells (**c**, ×200) surrounded by intact rim of myoepithelial cells (**d**, ADH5 ×200). Intraepithelial disease with focus of adjunct normal epithelium on right (**e**, ×100; **f**, ADH5 ×100)

The patient was referred to a hospital-based ENT service and, following CT imaging which did not identify an underlying soft tissue mass, and multidisciplinary tumor board discussion, a composite resection of involved mucosa and anterior mandible was performed (Fig. 4a). Intraoperative margin assessment showed focal margin involvement of clinically normal-appearing squamous mucosa by pagetoid cells (Fig. 4b), which was superseded by revised margins free of disease resulting in negative circumferential mucosal margins. The histologic findings of the resection specimen were essentially identical to those of the biopsy but showed, additionally, numerous underlying salivary excretory ducts involved, and occasionally significantly distended, by tumor cells (Fig. 4c–f). All unattached mucosa was entirely submitted and serially sectioned in attempt to identify an underlying occult primary malignancy, but the adenocarcinoma was exclusively intraepithelial/intraductal, with no invasive adenocarcinoma identified. This was further supported by multiplex immunohistochemistry for ADH5, which highlighted intraepithelial/intraductal Paget cells with CK7/18 and an intact myoepithelial layer around the intraductal component with CK5/14 and p63 (Fig. 5a–f). The case was again discussed at multidisciplinary tumor board, with recommendation for close follow-up at this time. The patient is well 4 months after surgery.

## Discussion

EMPD is exceptionally rare in the oral mucosa, with only nine cases having been reported in the English-language literature, including the present case (Table 1) [6–13]. EMPD has been reported in six males and three females at a median age of 65.5 years old. Clinically, all patients presented with variably erythematous, ulcerated or keratotic lesions bearing resemblance to lichen planus, with an identifiable mass usually absent. Mucosal involvement is typically extensive, ranging from 3.0 to 12.0 cm when measured and sometimes demonstrating pan-mucosal involvement. Nearly any oral mucosal subsite may be involved, with one case documenting perioral skin involvement as well. The extensive presentation of oral EMPD, mimicking an inflammatory process, is comparable to the presentation of PD at mammary and other extramammary sites [2].

**Table 1:** Clinical characteristics of oral EMPD

	Age (y)/Sex	Site	Clinical presentation	Size (cm)	Intraductal component	Invasive adenocarcinoma	Surgery	RTx	CTx	Recurrence (mos)	DOD (y)
Changus, et al	64/M	Tongue	Nipple-like protrusion	-	-	Y	Radical neck resection	Y	N	Y (-)	-
Theaker	67/M	Tongue, buccal, palate, oropharynx, glossopharyngeal sulcus	Extensive redness and focal ulceration	12.0	Y	Y	En bloc mucosal resection, bilateral radical neck dissection	Y	N	Y (6)	Y (1.5)
Kikuchi, et al	46/M	Submandibular gland, FOM, mandibular gingiva and buccal mucosa	Painful palpable mass under the FOM, erythematous mucosa	-	Y	Y	Submandibular gland resection and mucosal resection, bilateral upper neck dissection	Y	Y	Y (48)	Y (10)
Kennedy, et al	42/M	Hard palate, buccal gingiva	Florid red-white surface lesion	-	Y	-	Wide local excision of hard palate, facial maxillary gingiva, and bilateral buccal mucosa, with extraction of maxillary dentition	N	N	-	-
Wang, et al	55/F	Buccal and alveolar mucosa, palate, mandibular gingiva, perioral skin	Erythematous, erosive lesions, eczema-like with white scales	Pan-mucosal	-	-	None	N	N	N (36)	-
Molina, et al	50 s/F	Upper vermillion and cutaneous lip	Erythematous plaque	-	-	Y	Subtotal mucosal resection	Y	Y	Y	-
Shimomura, et al	86/F	Hard palate	Lichenoid lesions	4.5	Y	N	Radical resection of hard palate	N	N	Y (19)	-
Xue, et al	72/M	Buccal mucosa	Ulcer, white plaque	3.0	N	N	Wide local mucosal excision	N	N	N (12)	-
Present case	72/M	Buccal and lingual gingiva, FOM, labial mucosa	Red-white surface lesion	8.2	Y	N	Labial and FOM mucosa resection, segmental mandibulectomy	N	N	N (4)	N

*Data not reported is marked with a hyphen

Histologically, underlying invasive adenocarcinoma was documented in four cases of oral EMPD (diagnosed as anaplastic adenocarcinoma, salivary duct carcinoma, adenocarcinoma not otherwise specified, and poorly differentiated carcinoma). MPD is almost uniformly associated with underlying breast carcinoma, with nipple/areolar involvement representing extension from ductal carcinoma in situ (DCIS) involving underlying lactiferous ducts. EMPD, on the other hand, is accounted for by extension from an underlying primary cutaneous adnexal carcinoma in only approximately 25% of cases [5]. A further 10–15% of patients have carcinoma involving gastrointestinal or genitourinary sites with pagetoid spread of tumor cells into mucocutaneous surfaces of the anogenital region, resulting in a disease presentation classified as secondary EMPD. For the majority of cases of EMPD, however, the pathogenesis remains unclear.

It has been hypothesized that these cases of EMPD as well as rare cases of MPD not associated with underlying breast carcinoma may originate from Toker cells (clear cells of Toker), clear cells occasionally identifiable in the basal layer of squamous epithelium of the nipple and vulva [14, 15]. Toker cells (TC) bear some morphologic resemblance to Paget cells, as well as CK7 immunopositivity, and have been postulated to represent potential precursor cells to PD, though their exact origin and function remain unknown [16]. TC have historically been suggested to represent abortive mammary differentiation or intraepithelial extension of lactiferous duct cells but more recent studies have demonstrated localization of TCs to underlying sebaceous glands instead of to lactiferous ducts [17, 18]. If TCs are indeed related to underlying folliculo-sebaceous/apocrine units instead of to lactiferous ducts, this could potentially help to understand the occurrence of purely intraepithelial PD in the absence of underlying carcinoma, as well as the occurrence of EMPD outside of the milk line, including oral mucosa where sebaceous glands (Fordyce granules) may be present. However, the pathogenesis of primary EMPD remains speculative.

The management of oral EMPD is primarily surgical, frequently complicated by local or occasionally regional recurrence, as is the case for EMPD in general [19]. Tendency for local recurrence is interpreted in light of propensity of pagetoid cells to spread laterally beyond clinically visible disease, as was noted during frozen section examination of the present case. Local recurrence was documented in 5/9 patients with oral EMPD, one of which also demonstrated regional recurrence [6], over periods of 6–48 months. All four patients with invasive carcinoma also underwent radiation therapy and two of these patients also received chemotherapy. The two patients with oral EMPD and an invasive adenocarcinoma component died of disease after periods of 1.5 years and 10 years. One patient with pan-mucosal disease who was not a candidate for surgery and who declined radiation and chemotherapy was administered thalidomide [10]. This patient demonstrated reduction in lesion size over 5 months of therapy and clinically stable disease over 36 months follow-up.

Oral epithelial dysplasia (OED) and melanoma in situ (MIS) are two important differential diagnoses for EMPD. OED, MIS, and EMPD all present as an intraepithelial proliferation of cytologically atypical cells, and EMPD may show surface keratinization, as in this case, further mimicking OED. Accurate diagnosis requires immunohistochemical assessment, which should be considered whenever encountering enlarged, pleomorphic cells with abundant amphophilic cytoplasm. Immunohistochemically, ductal differentiation in EMPD can be demonstrated by expression of low-molecular weight cytokeratins (Cam5.2, CK7), CEA, EMA, AR, and Her2. Conventional squamous and melanoma markers such as CK5/6, p40, S100, and SOX10 are consistently negative (Table 2). It is important to highlight that TRPS1 immunohistochemical marker is commonly expressed in breast carcinoma, MPD and EMPD, as the present case demonstrates, but has been reported to be positive in cutaneous carcinoma in situ as well [20]. More work is needed to determine whether TRPS1 is expressed in OED and whether this may represent a diagnostic pitfall in this setting.

**Table 2:** Commonly reported immunohistochemical findings in oral EMPD

	CK7	Cam5.2	EMA	CEA	CK5/6	p63/p40	S100	Her2	AR
Changus, et al	-	-	-	-	-	-	-	-	-
Theaker	-	Pos	Pos	-	-	-	-	-	-
Kikuchi, et al	-	Pos	Pos	Pos	-	Neg	Neg	3 +	Pos
Kennedy, et al	-	-	-	-	-	-	-	-	-
Wang, et al	Pos	Pos	Pos	Pos	Neg	Neg	Neg	-	-
Molina, et al	-	-	-	-	-	-	-	-	-
Shimomura, et al	Pos	-	-	-	Neg	Neg	Neg	-	Pos
Xue, et al	Pos	-	Pos	Pos	Neg	Neg	Neg	1 +	-
Present case	Pos	Pos	Pos	Pos	Neg	Neg	Neg	3 +	Neg
Total	100% (4/4)	100% (4/4)	100% (5/5)	100% (4/4)	0% (0/4)	0% (0/5)	0% (0/5)	100% (3/3)	66.6% (2/3)

^*^Pos denotes positive; Neg denotes negative; hyphen denotes stain not reported/performed

A recently sequenced clinical cohort of vulvar EMPD identified targetable alterations in *PIK3CA* or *ERBB2* in > 25% of patients, raising the possibility of novel therapeutic approaches, though whether these alterations occur in oral EMPD remains to be validated [21]. *TP53* mutations were also identified in a subset of that cohort, as was identified in the present case by p53 IHC as diffuse nuclear overexpression in Paget cells. p53-mutant IHC may be a pitfall in the differential diagnosis with OED, in which patterns of p53-mutant immunoexpression have been recently described, including diffuse overexpression in tumor cells, null expression and, rarely, cytoplasmic expression in tumor cells [22]. Careful histologic and immunohistochemical evaluation is necessary for the diagnosis of oral EMPD.

In the present case, extensive involvement of multiple salivary ducts was documented by ADH5 IHC, which demonstrated the intraductal CK7/18 + Paget cells to be surrounded by an intact layer of CK5/14 + and p63 + myoepithelial cells, with no evidence of invasive adenocarcinoma. The involvement of multiple salivary ducts in the present case was interpreted to favor surface origin followed by colonization of multiple salivary ducts, though such a determination remains academic. It is difficult to rule out the possibility of origin within one salivary duct followed by pagetoid surface involvement and then subsequent colonization of adjacent salivary ducts. Intraductal involvement in oral EMPD is not uncommon, having been reported in five cases total, including the present case.

In conclusion, EMPD has been documented exceptionally rarely in the oral mucosa and diagnosis requires familiarity with the diagnosis as well a high index of suspicion in light of significantly more commonly occurring conditions such as lichenoid mucositis and oral epithelial dysplasia. More work is needed to identify targetable alterations in oral EMPD as well as to understand the pathogenesis of cases not associated with an underlying primary adenocarcinoma.

## Data Availability

No datasets were generated or analysed during the current study.
